# Investigating behavioral phenotypes related to autism spectrum disorder in a gene-environment interaction model of *Cntnap2* deficiency and Poly I:C maternal immune activation

**DOI:** 10.3389/fnins.2023.1160243

**Published:** 2023-03-14

**Authors:** Faraj L. Haddad, Cleusa De Oliveira, Susanne Schmid

**Affiliations:** ^1^Department of Physiology and Pharmacology, Schulich School of Medicine and Dentistry, The University of Western Ontario, London, ON, Canada; ^2^Department of Anatomy and Cell Biology, Schulich School of Medicine and Dentistry, The University of Western Ontario, London, ON, Canada; ^3^Department of Psychology, The University of Western Ontario, London, ON, Canada

**Keywords:** maternal immune activation, *Cntnap2*, Poly I:C, startle, pre-pulse inhibition, autism spectrum disorder, rat

## Abstract

**Introduction:**

Autism Spectrum Disorder (ASD) has been associated with a wide variety of genetic and environmental risk factors in both human and preclinical studies. Together, findings support a gene-environment interaction hypothesis whereby different risk factors independently and synergistically impair neurodevelopment and lead to the core symptoms of ASD. To date, this hypothesis has not been commonly investigated in preclinical ASD models. Mutations in the Contactin-associated protein-like 2 (*Cntnap2*) gene and exposure to maternal immune activation (MIA) during pregnancy have both been linked to ASD in humans, and preclinical rodent models have shown that both MIA and *Cntnap2* deficiency lead to similar behavioral deficits.

**Methods:**

In this study, we tested the interaction between these two risk factors by exposing Wildtype, *Cntnap2^+/–^*, and *Cntnap2*^–/–^ rats to Polyinosinic: Polycytidylic acid (Poly I:C) MIA at gestation day 9.5.

**Results:**

Our findings showed that *Cntnap2* deficiency and Poly I:C MIA independently and synergistically altered ASD-related behaviors like open field exploration, social behavior, and sensory processing as measured through reactivity, sensitization, and pre-pulse inhibition (PPI) of the acoustic startle response. In support of the double-hit hypothesis, Poly I:C MIA acted synergistically with the *Cntnap2*^–/–^ genotype to decrease PPI in adolescent offspring. In addition, Poly I:C MIA also interacted with the *Cntnap2^+/–^* genotype to produce subtle changes in locomotor hyperactivity and social behavior. On the other hand, *Cntnap2* knockout and Poly I:C MIA showed independent effects on acoustic startle reactivity and sensitization.

**Discussion:**

Together, our findings support the gene-environment interaction hypothesis of ASD by showing that different genetic and environmental risk factors could act synergistically to exacerbate behavioral changes. In addition, by showing the independent effects of each risk factor, our findings suggest that ASD phenotypes could be caused by different underlying mechanisms.

## Introduction

In recent decades, the rise of Autism Spectrum Disorder (ASD) prevalence has prompted many studies into its underlying etiology. In the field of genetics, the rapid advancement of DNA sequencing and genetic techniques has led to the discovery of many rare and common genetic variants associated with ASD (Manoli and State, 2021). Following a similar trend, various environmental factors that can influence neurodevelopment and increase the risk for ASD have been identified (Modabbernia et al., 2017). However, neither genetic nor environmental risk factors alone can account for all cases of the disorder, providing support for the gene-environment interaction hypothesis (Chaste and Leboyer, 2012). In this hypothesis, genetic and environmental risk factors produce a combined effect that alters prenatal and early postnatal brain development and lead to the hallmark ASD behavioral features of altered social interaction, communication, and repetitive behavior. Unfortunately, our current understanding of gene-environment interactions in the pathophysiology of ASD is limited, as studies have traditionally focused on studying individual risk factors (Varghese et al., 2017).

Beyond the complex etiology, deciphering precisely how genetic or environmental insults lead to the behavioral features of ASD has proven difficult. Although many advances have been made in developing ASD-related behavioral tests in preclinical models, it is still difficult for these models to replicate the human repertoire of social interaction, communication, and restricted or repetitive patterns of behavior (Crawley, 2012). To circumvent these limitations, one potential solution is to study basic sensory processing mechanisms that are more directly translatable between species. This avenue has been explored more closely in recent years, following the publication of the fifth edition of the Diagnostic and Statistical Manual of Mental Disorders (DSM V). In this new edition, the DSM emphasized hypo- or hyperreactivity to sensory input as a feature that can be associated with restricted, repetitive patterns of behavior, interests, or activities (American Psychiatric Association, 2013). Similarly, altered sensory processing can be linked with social behavior and communication, which often require integrating large amounts of sensory information (Thye et al., 2018).

In this study, our primary goal was to study sensory processing behaviors in a multifactorial gene-environment interaction rodent model of ASD. The two risk factors in our model were maternal immune activation (MIA) during pregnancy and a genetic deficiency in Contactin associated protein-like 2 (*Cntnap2*). A homozygous loss-of-function mutation in the human *CNTNAP2* leads to a syndromic form of ASD, with the syndrome also including cortical dysplasia, seizures, language regression, and cognitive delays (Strauss et al., 2006; Jackman et al., 2009; Zweier, 2011; Peñagarikano and Geschwind, 2012; Rodenas-Cuadrado et al., 2016). These findings prompted the development of *Cntnap2* knockout animal models to study ASD-related pathophysiology. On the other hand, exposure to various types of maternal infection during pregnancy is known to increase the offspring’s risk of developing ASD, which implicates the maternal immune response, a common factor between different infections, as a disruptor of neurodevelopment (Jiang et al., 2016; Haddad et al., 2020b). Following these epidemiological associations, animal models were developed to investigate how MIA, induced through injection of an immune stimulant at various times during pregnancy, impacts neurodevelopment and postnatal phenotypes.

Preclinical studies investigating the effects of *Cntnap2* knockout and MIA have shown strong face validity in relation to ASD and potential for interaction. For example, both *Cntnap2*^–/–^ mice and MIA offspring show deficits in social behavior, increased repetitive behavior, and increased sensory reactivity (Peñagarikano et al., 2011, 2015; Brimberg et al., 2016; Richetto et al., 2017; Vuillermot et al., 2017; Weber-Stadlbauer et al., 2017; Hui et al., 2018; Scott et al., 2018, 2020; Haddad et al., 2020a). Furthermore, a study by Schaafsma et al. (2017) combined MIA and *Cntnap* knockout in mice and demonstrated a sex-specific gene-environment interaction in ultrasonic vocalizations, social behavior, and locomotor hyperactivity. Beyond behavioral phenotypes, both Poly I:C MIA offspring and *Cntnap2*^–/–^ mice show similar cellular and molecular brain phenotypes including disorganized cortical layering (Peñagarikano et al., 2011; Choi et al., 2016; Coutinho et al., 2017), decreased expression of Parvalbumin in the hippocampus (Abazyan et al., 2010; Peñagarikano et al., 2011; Lipina et al., 2013; Brunner et al., 2015), decreased cortical and hippocampal dendritic arborization (Li et al., 2014; Brimberg et al., 2016; Fernandes et al., 2019; Xu et al., 2019), and increased microglial activation (Coutinho et al., 2017; Hui et al., 2018).

This study sought to explore the *Cntnap2*-MIA interaction in the context of sensory processing, as measured by reactivity, sensitization, and pre-pulse inhibition (PPI) of the acoustic startle response. These startle phenotypes are also measurable in humans and provide a direct comparison to human ASD studies (Kohl et al., 2014; Takahashi et al., 2017). In addition, our study utilized a different MIA protocol with Polyinosinic: polycytidylic acid (Poly I:C) as the immunogen of choice, injected at gestation day (GD) 9.5, which is one of the most commonly used protocols in the MIA literature (Haddad et al., 2020b). Finally, we were interested in determining whether MIA would interact with a partial *Cntnap2* deletion in heterozygous animals, which typically resemble Wildtype animals (Scott et al., 2020).

## Materials and methods

### Animals

This study was conducted using Wildtype (WT), *Cntnap2* heterozygous (*Cntnap2^+/–^*) and homozygous (*Cntnap2*^–/–^) knockout rats on a Sprague Dawley background. The *Cntnap2* rat model contains a 5 base pair frameshift deletion in exon 6 of the *Cntnap2* gene, resulting in total functional loss of the *Cntnap2* protein CASPR2, as confirmed via Western blot (ENVIGO). Original breeders were purchased from ENVIGO *in vivo* services and then licensed for in-house breeding. In this experiment, genotyping was performed using genomic DNA collected at weaning. The genomic DNA was amplified using PCR and then sequenced to detect the 5-base-pair deletion in *Cntnap2^+/–^* and *Cntnap2*^–/–^ offspring (see Supplementary Figure 1 for representative sequencing results). All animal procedures were approved by the Western Animal Care Committee and adhered to the guidelines of the Canadian Council on Animal Care.

Rats were housed in open cages with sawdust bedding, given *ad libitum* food and water, and kept on a 12 h light–12 h dark cycle with lights turning on at 7:00 am. Cages were enriched with polycarbonate huts and wrinkled paper. Cage changes took place once a week except during behavioral testing procedures, during which cage changes were carried out at the end of the 5-day startle protocols to ensure startle measures were not influenced by cage change stress. All behavioral testing and cage changes took place during the light phase (between 7:00 and 19:00 h).

### Timed breeding

We used the same male rat to generate all 18 litters in this study over 6 months to minimize the impact of variation in paternal characteristics and ensure our results can be more specifically linked to the maternal immune response. In each breeding round, the *Cntnap2^+/–^* breeding male (aged 6 months at the start of the experiment and 12 months by the end of the experiment) was paired with 2–3 *Cntnap2^+/–^* females (aged 3–9 months). Additionally, one litter in this experiment was accidentally generated by pairing a *Cntnap2*^–/–^ female with the *Cntnap2^+/–^* male. Statistical inspection of behavioral data revealed that offspring from this litter were not different from those obtained by full heterozygous breeding and were therefore included in all the results. Breeding rounds were at least 1 week apart. There were six breeding rounds in total. Five of these rounds contained at least one saline and one Poly I:C dam. At the end of these five rounds, the WT saline offspring group was the smallest, so a sixth breeding round containing only saline-treated dams with three females was performed to increase the sample size for this group. The breeding females across all breeding rounds originated from four different genetic families (4–5 females per family) that were all unrelated to the breeding male.

After pairing, a vaginal smear was collected from each female at 8 a.m. every morning and inspected under a light microscope to track the estrus cycle and check for the presence of sperm. If sperm was detected in the smear, the female was considered pregnant, and that day was considered Gestation Day (GD) 0.5. Pregnant females were separated from the male on GD0.5 and transferred into a single cage, where they were left undisturbed until injection day (GD9.5). All breeding females were primiparous at the start of the experiment. Only one female was bred twice in this experiment, all other females were bred only once. For the female that was bred twice, visual inspection of startle and open field behavioral data, stratified by age, sex, and genotype, confirmed that the second litter showed similar phenotypes to primiparous litters in the same experimental group.

### Maternal immune activation

Pregnant females were randomly assigned to receive either Poly I:C or saline injections. MIA was induced using Poly I:C (Sigma lot #118M4035V), which had been previously aliquoted and stored at −20°C. Poly I:C aliquots were diluted in 0.9% saline to obtain a concentration of 4 mg/ml. At around 10 a.m. on GD9.5, pregnant females were weighed and had their rectal temperature measured. Then, they underwent isoflurane anesthesia (5% induction, 2% maintenance), during which they were injected with either 0.9% saline or 4 mg/kg of Poly I:C into the tail vein. The injection procedure took an average of 5 min and rats were returned to their cages afterward and were left undisturbed besides blood collection, temperature/weight measurement, and weekly cage change. Temperature measurements were taken 3-, 24-, and 48- h following injection, whereas maternal weight was recorded 24- and 48- h following injection. Similar to our previous Poly I:C MIA experiment (Haddad et al., 2020a), we found no significant differences in post-injection temperature and weight gain data between the Poly I:C and saline dams (data not shown). Since body temperature and weight gain following Poly I:C MIA seem to be unreliable indicators of the maternal immune response (Haddad et al., 2020b), we confirmed the immune response by measuring the level of proinflammatory cytokines in the maternal circulation.

On the day of injection, maternal blood was collected from the dams 3 h following injection. Under isoflurane anesthesia, 0.5–1 mL of blood was drawn from the tail vein and stored in serum separation tubes (SARSTEDT Microvette). The blood was allowed to clot for 30 min at room temperature and then spun at 2000 g and 4°C for 10 min. Serum was then collected and stored at −20°C to later be used in the ELISA cytokine experiment to assess cytokine expression.

The day of parturition was designated as postnatal day (PND) 0. Offspring were weaned at PND21, and littermates were separated based on sex (but not genotype) and housed in groups of 2–4 rats per cage. This resulted in the cohousing of sex-matched littermates possessing different *Cntnap2* genotypes. At the end of the experiment, the final number of litters in the saline group was as follows: Ten litters were born from saline dams, and eight litters were born from Poly I:C dams.

### Quantification of the immune response—Enzyme-linked immunosorbent assay (ELISA)

Post-injection serum was used to quantify the maternal immune response to Poly I:C using DuoSet Interleukin (IL)-6 (Catalog# DY506; Lot P216627; R&D Systems) and Tumor Necrosis Factor (TNF)-α (Catalog# DY510; Lot P237489; R&D Systems) ELISAs supplemented with the ELISA ancillary kit (Catalog# DY008B; LotP240500; R&D Systems). The procedure was conducted according to the manufacturer’s instructions with a couple of modifications. Due to the presence of matrix effects in the serum that could disrupt the signal produced by the cytokines of interest, samples were diluted 10-fold as opposed to the twofold dilution recommended by the Duoset instruction manual (10% serum, 90% buffer solution). Additionally, 10% control serum was obtained from a control rat to establish the cytokine standards used to generate the standard curve, so that the curve is representative of a signal containing 10% serum and would help more accurately determine the true concentration of IL-6 in the samples. A standard curve was generated using linear regression of the log concentration plotted against the log optical density for positive and negative controls. This curve was then used to interpolate cytokine concentrations in the serum samples.

### Offspring testing outline

**FIGURE 1:**
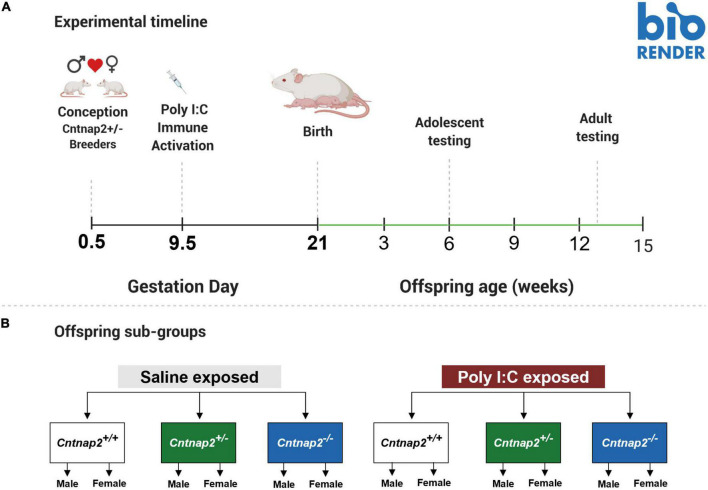
Summary of experimental timeline and groups. **(A)** Illustration showing the experimental timeline, including the timing of MIA and behavioral experiments. **(B)** A schematic showing the different MIA and genotype subgroups. There were 6 MIA-genotype groups, and 12 total subgroups when accounting for sex.

Startle, open field, and social behavior testing occurred in adolescence and adulthood. Adolescent testing began around PND38-39, and adult testing began around PND90-100. Animals were weighed and behaviorally tested in both adolescence and adulthood, with the whole battery of tests taking 9 days to complete at each age timepoint. Tests were performed in the following order: Startle acclimation and handling (Day 1), social behavior (Days 2 and 3), open field and startle reactivity (Day 4), startle sensitization (Days 5–8), and PPI (Day 9). To get animals acclimated to the experimenter, adolescent animals were handled at least twice, 3–5 min each time, before startle acclimation. The full experimental timeline is shown in Figure 1.^1^

### Acoustic startle response

#### Acclimation

Startle testing was performed as described by Valsamis and Schmid (2011). After handling, rats were initially acclimated to the experimental procedure, Plexiglas animal holders, and startle chambers (Med Associates, VT, USA) by undergoing three 5-min acclimation sessions, at least 6 h apart, with 65 dB sound pressure level (SPL) white noise background. The Plexiglass animal holders were washed with unscented soap between acclimation runs, and the same washing procedure was used for all the startle testing described below.

Startle Reactivity After the acclimation sessions, startle reactivity in response to startle stimuli of varying intensities was measured. This test consisted of white noise startle stimuli at intensities ranging from 65 to 120 dB in 5 dB increments, presented in a pseudorandomized order. The startle reactivity session used a constant platform sensitivity for all animals. Based on data from this test, platform sensitivity was then adjusted for each rat to optimally detect the startle reflex in all remaining startle tests.

#### Sensitization

Animals were exposed to a 5-min acclimation period with background noise, followed by a block of 30 trials. In this block, each trial consisted of a 20 ms-long, 100 dB white noise stimulus after which the startle amplitude was measured. A relatively long ITI was used (1 min) to minimize short-term habituation effects, thereby exposing sensitization effects (Azzopardi et al., 2018). To compare sensitization between groups, sensitization curves for day 1 of startle testing were generated, depicting the startle amplitude for each of the 30 trials in the session. However, these curves showed that animals across all groups showed a large degree of between-trial variability. Therefore, we decided to bin all trials following trial 1 into bins of 3 or 4 trials. Bin 1 was the average of the first 4 trials after trial 1 (trials 2–5), whereas bin 9 was the average of the last 4 trials in the session (trials 27–30). Bins 2–8 each consisted of the average of 3 trials and encompassed trials 6–26. Finally, a sensitization score (Bin 1 / Trial 1) was calculated for each animal, which produced a normalized value that accounted for each animal’s baseline startle at trial 1.

#### PPI

The PPI session consisted of an initial block of 20 trials (110 dB, 20 ms duration), followed by a PPI block. In the PPI block, animals were exposed to either startle-only trials (110 dB, 20 ms duration), or pre-pulse trials, which included a 4 ms white noise pre-pulse of either 75, 80, or 85 dB preceding the startle stimulus by an interstimulus interval (ISI) of either 30 or 100 ms. ISI was determined as the time between the onset of the pre-pulse to the onset of the startle stimulus. In total, the 60 pre-pulse trials were divided into 10 of each of the following pre-pulse-ISI combinations: 75–30, 75–100, 80–30, 80–100, 85–30, and 85–100. The trials were presented in a pseudorandomized order and separated by a 12–18 s randomized ITI. After the presentation of the startle stimulus in each startle-only or pre-pulse trial, the startle amplitude was measured. PPI was calculated as the amount of inhibition of startle in pre-pulse trials compared to startle-only trials in the PPI block:


$$\%PPI=\left[1-\left(\frac{a\,v\,e\,r\,a\,g\,e\,s\,t\,a\,r\,t\,l\,e\,m\,a\,g\,n\,i\,t\,u\,d\,e\,w\,i\,t\,h\,p\,r\,e\,p\,u\,l\,s\,e}{a\,v\,e\,r\,a\,g\,e\,s\,t\,a\,r\,t\,l\,e\,w\,i\,t\,h\,o\,u\,t\,p\,r\,e\,p\,u\,l\,s\,e\,i\,n\,P\,P\,I\,b\,l\,o\,c\,k}\right)\right]\times 100.$$

### Open field test

The day following the PPI test, rats were placed in a square open field of 45.7 cm × 45.7 cm dimension for 30 min as described previously (Fulcher et al., 2020). This test was used to determine locomotor activity by measuring the total distance traveled throughout the test. Additionally, anxiety-like behavior was measured through the time spent in the center of the chamber. The animal’s head location was monitored by an overhead camera as they freely explored the box; the data was collected and analyzed using ANYMAZE software (V4.99, Stoelting, Wood Dale, IL, USA). The apparatus was cleaned with 70% ethanol between experiments.

### Social behavior

Each rat’s preference for a conspecific over an inanimate object (sociability), as well as its preference for a stranger rat over a familiar rat (social novelty), were assessed using the previously established 3-chamber assay (Moy et al., 2004; Crawley, 2007; Scott et al., 2020).

The apparatus was 115 cm long and 58 cm wide, with transparent walls that were 45 cm high. The apparatus was split equally into 3 chambers, and two gates (10 cm wide) separated the center chamber from the left and right side chambers. The gates were always open, and the animal was free to explore and move between chambers at all points during the test. In the middle of each of the side chambers, a plexiglass animal holder that allowed nose contact was placed.

The test began by placing the test rat in the center chamber, with empty animal holders in the left and right chambers. The test rat was given 10 min to explore and habituate to the entire apparatus. Following habituation, sociability preference was determined by placing a stranger rat (stranger 1) in one of the side chambers and allowing the test rat to explore for 10 min. The stranger was a sex-matched adult animal between 3 and 6 months of age. The strangers were housed in the same holding room as the experimental animals. To account for potential side bias that animals sometimes exhibit in this kind of test, the side where stranger 1 was placed was chosen randomly, such that roughly half of the animals had stranger 1 placed in the left chamber, and the other half had stranger 1 placed in the right chamber. Then, social novelty preference was determined by placing a novel stranger rat (stranger 2) in the opposing side chamber and allowing the test rat to explore for 10 min. During social novelty, stranger 1 is considered familiar to the test rat, and stranger 2 is considered novel. The test rat was not removed from the apparatus while placing the strangers into the side chambers to avoid additional stress, which can alter social and exploratory behavior. Between different test rats, the animal holders were washed with unscented soap, and the entire apparatus was wiped with 70% ethanol.

The test rat’s exploration was tracked using a camera and its activity in each chamber was quantified using ANYMAZE software (V4.99, Stoelting, Wood Dale, IL, USA). Additionally, the animal’s close exploration (sniffing) of the animal holders was tracked, which was specified as the animal’s nose (as detected by ANYMAZE) being within 3 cm of the animal holder.

### Statistical analysis

#### Maternal serum cytokines

A one-tailed independent *t*-test was conducted for the levels of circulating proinflammatory cytokines (measured in pg/mL for both IL-6 and TNF-α) comparing 8 saline and 8 Poly I:C dams. These tests were adjusted to account for the violation of the assumption of equal variance since there was more variability in the levels of serum cytokines in Poly I:C dams compared to saline dams.

#### Offspring behavioral data

Before conducting analysis, outliers were systematically removed to exclude extreme values and to allow the data distributions to better approximate a normal distribution that is required for the parametric statistical tests described later in this section.

Data were scanned using the SPSS Explore function on each genotype-MIA-sex group in each age time point to detect outliers (for each age time point, 12 groups in total: 3 genotypes x 2 MIA groups × 2 sexes). Within each genotype-MIA-sex group, outliers were defined as either moderate or extreme: Moderate outliers were those that had a value of 1.5 interquartile ranges above the 75th percentile or below the 25th percentile; Extreme outliers were those that had a value of 3 interquartile ranges above the 75th percentile or below the 25th percentile.

Within each test at each age time point (open field, sociability, social novelty, startle), animals that were extreme outliers in any of the different measures or were moderate outliers in two or more measures were excluded from the data analysis for that test at that age time point. These exclusion criteria were chosen because many measures within each test are interdependent (e.g., an animal’s locomotor activity influences that probability of them spending time in the center of the open field).In the startle tests, additional outlier exclusion was conducted for separate measures (reactivity, sensitization curves, sensitization scores, PPI), as these measures were conducted on separate days and were influenced by trial-to-trial variability. For example, some animals that did not startle on trial 1 in the sensitization sessions showed extremely high sensitization scores that are not representative of their typical behavior or their respective groups. These animals were excluded for sensitization score statistical analysis, but not necessarily for the PPI statistical analysis, given that PPI was conducted on a separate day when these animals showed values comparable to their respective groups. This resulted in slightly different sample sizes for different startle measures.

The final animal numbers per group for each test at each age time point are summarized in Table 1. For each behavioral test, a three or four-way analysis of variance (ANOVA) was conducted separately for adolescent and adult data for each dependent variable. In the startle test, the dependent variables were startle reactivity (unit: arbitrary units), sensitization (no unit, normalized value), and PPI (no unit, normalized value). In the open field test, the dependent variables were total distance traveled in the 30 min session (unit: meters) and the total time spent in the center of the open field (unit: seconds). In the social behavior test, the dependent variables were time spent sniffing the animal holders in the sociability stage (unit: seconds) and time spent sniffing the animal holders in the social novelty stage (unit: seconds). All ANOVAs included genotype, sex and MIA as between-subject factors. Additional within-subject factors were included based on the measure of interest: Stimulus intensity for startle reactivity, trial/bin number for sensitization curves, pre-pulse intensity and ISI for PPI, and animal holder side for social behavior. All *post-hoc* testing was adjusted using the Bonferroni adjustment for multiple comparison.

**TABLE 1:** The number of offspring per group for each behavioral experiment after outlier exclusion.

Group	Age	Startle reactivity Figure 3	Sensitization curves Figures 4A,C,E,G	Sensitization scores Figures 4B,D,F,H	PPI Figure 5	Open field Figure 6	Sociability Figures 7A,B	Social novelty Figures 7C,D
**Wildtype**								
Saline	6 weeks	8 (6)	8 (6)	8 (6)	6 (4)	9 (6)	10 (6)	9 (6)
Male	3 months	8 (6)	8 (6)	7 (6)	8 (6)	9 (6)	10 (6)	9 (5)
Poly I:C	6 weeks	8 (6)	8 (6)	8 (6)	8 (6)	9 (6)	8 (5)	8 (6)
Male	3 months	8 (6)	8 (6)	8 (6)	8 (6)	8 (6)	8 (5)	6 (4)
Saline	6 weeks	11 (4)	11 (4)	11 (4)	10 (4)	14 (6)	14 (6)	13 (6)
Female	3 months	11 (4)	11 (4)	10 (4)	10 (4)	11 (6)	12 (5)	10 (4)
Poly I:C	6 weeks	8 (4)	8 (4)	8 (4)	8 (4)	10 (5)	8 (5)	10 (5)
Female	3 months	8 (4)	8 (4)	8 (4)	7 (4)	9 (5)	9 (5)	9 (5)
***Cntnap2^+/–^***								
Saline	6 weeks	23 (8)	23 (8)	22 (8)	23 (8)	22 (8)	23 (8)	22 (7)
Male	3 months	21 (8)	21 (8)	17 (8)	20 (8)	20 (8)	21 (8)	20 (8)
Poly I:C	6 weeks	18 (6)	18 (6)	17 (6)	18 (6)	19 (6)	19 (6)	16 (6)
Male	3 months	17 (6)	17 (6)	16 (5)	16 (6)	17 (6)	18 (6)	17 (6)
Saline	6 weeks	24 (7)	24 (7)	23 (7)	24 (7)	23 (7)	25 (7)	23 (7)
Female	3 months	20 (7)	20 (7)	19 (7)	18 (7)	20 (6)	18 (7)	17 (7)
Poly I:C	6 weeks	14 (8)	14 (8)	14 (8)	13 (8)	15 (6)	13 (7)	16 (8)
Female	3 months	14 (8)	14 (8)	14 (8)	12 (7)	13 (7)	16 (8)	16 (8)
***Cntnap2* ^–/–^**								
Saline	6 weeks	17 (8)	17 (8)	17 (8)	17 (8)	18 (9)	19 (9)	18 (9)
Male	3 months	17 (8)	17 (8)	16 (8)	15 (7)	19 (6)	17 (9)	16 (9)
Poly I:C	6 weeks	15 (6)	15 (6)	14 (6)	14 (6)	16 (6)	14 (6)	16 (6)
Male	3 months	15 (6)	15 (6)	15 (6)	14 (6)	16 (6)	15 (6)	15 (6)
Saline	6 weeks	11 (7)	11 (7)	11 (7)	11 (7)	14 (7)	14 (7)	14 (7)
Female	3 months	11 (7)	11 (7)	10 (7)	11 (7)	14 (7)	12 (7)	14 (7)
Poly I:C	6 weeks	16 (7)	16 (7)	16 (7)	15 (6)	18 (7)	18 (7)	17 (7)
Female	3 months	14 (7)	14 (7)	13 (7)	14 (7)	15 (7)	14 (6)	15 (7)

Different experimental groups are shown in rows, whereas the different behavioral tests are shown in columns. Each cell contains the number of animals in the respective test and group. The number in brackets represents the number of different litters that the offspring originated from.

## Results

### Maternal serum cytokine response

**FIGURE 2:**
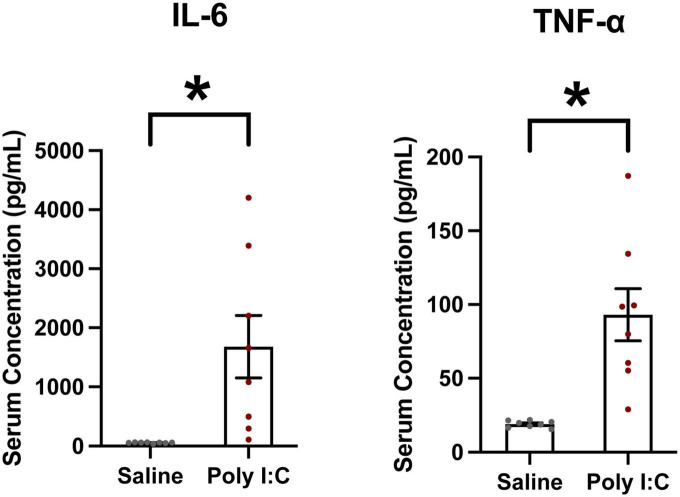
Poly I:C MIA significantly increases circulating levels of proinflammatory cytokines Interleukin-6 (IL-6) and Tumor Necrosis Factor α (TNFα) in maternal serum. Serum samples were collected 3 h following Poly I:C injection and quantified using ELISA. Both IL-6 and TNFα were significantly elevated in serum from Poly I:C-treated dams compared to saline-treated dams. Data are shown as mean ± standard error, *p < 0.05. Individual dots represent individual dams (*n* = 8 per group).

To confirm the efficacy of our Poly I:C MIA, the maternal serum cytokine response was measured 3 h post-injection. The proinflammatory cytokines of interest were IL-6 and TNFα, commonly elevated following Poly I:C exposure (Esslinger et al., 2016; Goeden et al., 2016; Mueller et al., 2019; Openshaw et al., 2019). There was a significant increase in serum IL-6 (*t* = 3.065; *df* = 7, *p* = 0.0091) and TNFα (*t* = 4.186; *df* = 7, *p* = 0.002) 3 h after Poly I:C administration, confirming that the protocol was successful in inducing a strong maternal immune response (Figure 2). Cytokine levels in saline dams were all just above the detection threshold of the ELISA. For both cytokines, data from Poly I:C dams were variable, but even the least responsive Poly I:C dam had an elevated level of IL-6 and TNFα compared to all saline dams (IL-6 highest saline dam–65 pg/mL; IL6 lowest Poly I:C dam–110 pg/mL; TNFα highest saline dam–21 pg/mL; TNFα lowest Poly I:C dam–29 pg/mL).

### Acoustic startle phenotypes

#### Reactivity to various stimulus intensities

**FIGURE 3:**
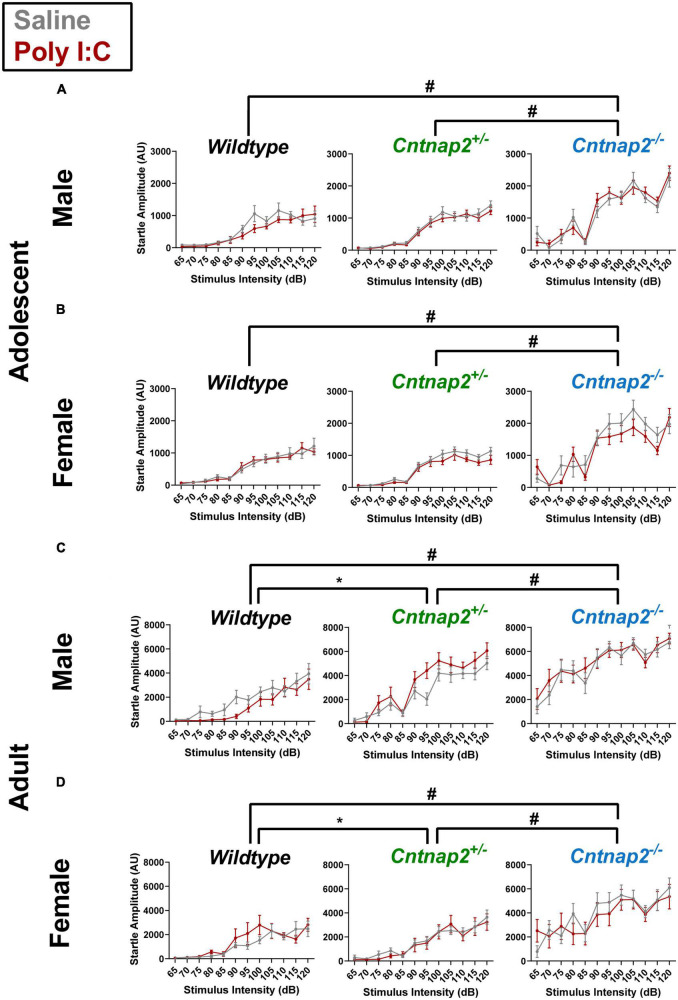
Partial and full *Cntnap2* knockout show age-dependent effects on startle reactivity. **(A,B)** *Cntnap2*^–/–^ adolescent animals show a general increase in startle reactivity compared to *Cntnap2^+/–^* animals at all stimulus intensities except at 70 and 85 dB (adjusted ^#^*p* < 0.05 for each stimulus intensity comparison). **(C,D)** In adulthood, *Cntnap2*^–/–^ offspring startled significantly higher than *Cntnap2^+/–^* and WT offspring at all intensities (^#^*p* < 0.05 for each stimulus intensity comparison), whereas *Cntnap2^+/–^* offspring startled significantly higher than WT offspring at 100 dB or higher intensities (adjusted *p < 0.05 for each stimulus intensity comparison). Data are shown as mean ± standard error. For information on sample sizes for each group, please see Table 1.

A separate repeated measures ANOVA was conducted at each age time point with stimulus intensity (65 to 120 dB in 5 dB steps) as a within subject factor and genotype, MIA, and sex as between subject factors. In adolescence, the ANOVA revealed a significant main effect of genotype [*F*_(2,161)_ = 77.791, *p* < 0.001] on startle response amplitudes, and a stimulus intensity*genotype interaction [*F*_(13.1,1053)_ = 10.096, *p* < 0.001]. Following *post-hoc* testing, *Cntnap2*^–/–^ offspring exhibited significantly increased startle reactivity compared to both *Cntnap2^+/–^* and WT offspring, regardless of MIA status, at all stimulus intensities except 70 and 85 dB (adjusted *p* < 0.05 for each significant comparison, indicated by the ^#^ symbol in Figures 3A, B).

In adulthood, there was also a significant main effect of genotype [*F*_(2,152)_ = 57.377, *p* < 0.001] and a significant stimulus intensity*genotype interaction [*F*_(15.0,1143)_ = 10.096, *p* < 0.001]. *Post-hoc* testing revealed that *Cntnap2*^–/–^ offspring startled more than *Cntnap2^+/–^* and WT offspring at all stimulus intensities (adjusted *p* < 0.05 for each significant comparison, indicated by the ^#^ symbol in Figures 3C, D). In addition, *Cntnap2^+/–^* offspring startled more than WT offspring at all decibels louder than 95 dB (adjusted *p* < 0.05 for each significant comparison, indicated by the * symbol in Figures 3C, D). Although the curves in Figures 3C, D suggest that males were influenced differently from females, this effect did not reach statistical significance in the form of a genotype*sex interaction [*F*_(2,152)_ = 1.890, *p* = 0.155] or a stimulus intensity*genotype*sex interaction [*F*_(15.03,1143)_ = 1.163, *p* = 0.295].

Overall, these data show that *Cntnap2*^–/–^ offspring, regardless of Poly I:C MIA or sex, exhibit increased startle reactivity to a broad range of stimulus intensities in both adolescence and adulthood. In addition, there is an intermediate reactivity phenotype in *Cntnap2^+/–^* offspring that manifests in adulthood.

#### Sensitization of startle upon repeated stimulation

A feature that makes the acoustic startle response a great measure of pre-attentive sensory processing is how it can be modulated in various conditions. In the case of repeated stimulation to the same startle stimulus, the response can either sensitize (increase) or habituate (decrease), with each of these processes having distinct cellular and molecular correlates (Koch, 1999). Therefore, the startle amplitude in every consecutive trial is a sum of an individual’s baseline reactivity, habituation, and sensitization during that particular trial.

One of the outstanding questions from previous experiments in both humans and preclinical models is whether the increase in startle amplitude is due to an increase in baseline reactivity or an increase in sensitization. Closer inspection of our data from Figures 3A, B indicated that sensitization is playing a role in our results. Specifically, we noted a marked decrease in the startle response to 85- and 115 dB trials in adolescent *Cntnap2*^–/–^ offspring. In our startle protocol, these 2 trials were presented earlier in the session compared to other trials of comparable stimulus durations and intensities (80 and 90 dB; 110 and 120 dB). To further investigate whether our startle reactivity phenotype was due to altered sensitization, we conducted a follow-up experiment where animals were exposed to 30 trials of 100 dB stimuli. We used an ITI of 1 min to minimize short-term habituation and therefore induce a robust sensitization effect. In addition, to account for inter-trial variability, we binned the trials after trial 1 into 9 separate bins of 3–4 trials each, with trial 1 representing the baseline reactivity and the different bins representing reactivity influenced by sensitization and habituation throughout the session.

**FIGURE 4:**
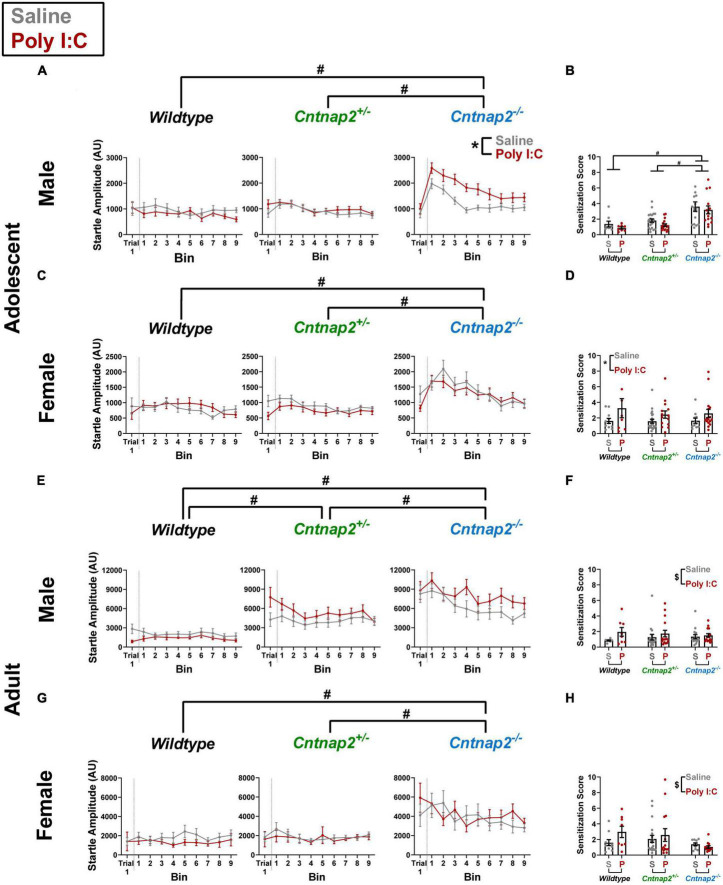
Independent and synergistic effects of *Cntnap2* knockout and Poly I:C MIA on sensitization of startle that manifest in an age, sex, and measurement-specific manner. **(A)** Adolescent *Cntnap2*^–/–^ males show increased startle reactivity compared to both *Cntnap2^+/–^* and WT males across the entire session (adjusted *p* < 0.001 for both comparisons; independent genotype effect indicated by the ^#^ symbol). In addition, *Cntnap2*^–/–^ Poly I:C males show increased startle reactivity compared to *Cntnap2*^–/–^ saline males (*p* < 0.001; synergistic genotype-MIA effect indicated by the * symbol). **(B)** Sensitization scores show that only the independent genotype effect remains after normalizing the baseline startle response amplitude (adjusted *p* < 0.001; indicated by the ^#^ symbol). **(C)** Adolescent *Cntnap2*^–/–^ females how increased startle reactivity compared to both *Cntnap2^+/–^* and WT females across the entire session (adjusted *p* < 0.001 for both comparisons; independent genotype effect indicated by the ^#^ symbol). **(D)** Adolescent Poly I:C females show increased sensitization scores compared to adolescent saline females regardless of genotype (*p* = 0.04; indicated by the * symbol). **(E)** Adult *Cntnap2*^–/–^ and *Cntnap2^+/–^* males both show increased startle reactivity compared to WT males across the entire session (adjusted *p* < 0.001 for both comparisons; independent genotype effect indicated by the ^#^ symbol). **(F)** Genotype does not alter sensitization scores in adult males, but Poly I:C offspring sensitize more than saline offspring regardless of sex and genotype (*p* = 0.047 indicated by the ^$^ symbol). **(G)** Adult *Cntnap2*^–/–^ females how increased startle reactivity compared to both *Cntnap2^+/–^* and WT females across the entire session (adjusted *p* < 0.001 for both comparisons; indicated by the ^#^ symbol). **(H)** Genotype does not alter sensitization scores in adult females, but Poly I:C offspring sensitize more than saline offspring regardless of sex and genotype (*p* = 0.047 indicated by the ^$^ symbol). Data are shown as mean ± standard error. For information on sample sizes for each group, please see Table 1.

A repeated measures ANOVA was conducted with trial 1/bins 1–9 as a within subject factor and genotype, MIA, and sex as between subject factors. In adolescence, there was a significant genotype*MIA*sex interaction [*F*_(2,161)_ = 3.453, *p* = 0.034] on startle response amplitudes. To follow up on this interaction, separate ANOVAs were conducted for each sex. In males, there was a significant genotype*MIA interaction [*F*_(2,83)_ = 5.308, *p* = 0.007]. *Post-hoc* testing revealed that Poly I:C *Cntnap2*^–/–^ males startled significantly higher throughout the session compared to saline *Cntnap2*^–/–^ males (*p* < 0.001; indicated by the * symbol in Figure 4A). This effect was not present in adolescent *Cntnap2*^–/–^ females (Figure 4C). In addition, both males and females showed a significant main effect of genotype, whereby *Cntnap2*^–/–^ offspring started significantly higher throughout the session compared to both *Cntnap2*^–/–^ and WT offspring (adjusted *p* < 0.001 for both comparisons; indicated by the ^#^ symbol in both Figures 4A, C).

In adulthood, there were no significant effects associated with Poly I:C MIA but there was a significant genotype*sex interaction [*F*_(2,152)_ = 5.747, *p* = 0.004]. *Post-hoc* testing revealed that both *Cntnap2*^–/–^ and *Cntnap2^+/–^* males startled significantly higher throughout the session compared to WT males (adjusted *p* < 0.001 for both comparisons; indicated by the ^#^ symbol in Figure 4E). In contrast, *Cntnap2^+/–^* females were not significantly different from WT females, and the only difference in female offspring was that of increased startle amplitude across the session in *Cntnap2*^–/–^ females compared to both *Cntnap2^+/–^* and WT females (adjusted *p* = 0.001 and 0.002, respectively; indicated by the ^#^ symbol in Figure 4G).

A challenge that arises when comparing sensitization curves between groups is that they do not properly account for each animal’s baseline reactivity, which can lead to false sensitization effects if animals of one group startled more intensely, as is the case for *Cntnap2* knockouts. It is known that the biggest sensitization effect is typically measured in early trials (Groves and Thompson, 1970). Therefore, we calculated a sensitization score by dividing each animal’s average startle amplitude in bin 1 by its amplitude in trial 1 and compared the resulting scores between groups.

A separate univariate ANOVA was conducted for the sensitization scores at each age with genotype, MIA, and sex as between subject factors. In adolescence, there was a significant genotype*sex interaction [*F*_(2,157)_ = 7.695, *p* = 0.001], a significant MIA*sex interaction [*F*_(1,157)_ = 9.1, *p* = 0.003], as well as a main effect of genotype [*F*_(2,157)_ = 6.583, *p* = 0.002]. *Post-hoc* testing showed that *Cntnap2*^–/–^ males sensitized significantly more than *Cntnap2^+/–^* (*p* < 0.001) and WT males (adjusted *p* < 0.001; indicated by the ^#^ symbol in Figure 4B), and this effect was absent in females (Figure 4D). Moreover, Poly I:C females sensitized significantly more than saline females regardless of genotype, and this effect was absent in males (*p* = 0.04; indicated by the * symbol in Figure 4D).

In adulthood, the ANOVA showed a significant main effect of Poly I:C MIA [*F*_(1,141)_ = 4.019, *p* = 0.047] where Poly I:C offspring showed higher sensitization compared to saline offspring regardless of genotype (indicated by the ^$^ symbol in Figures 4F, H). In contrast to adolescent data, there were no genotype-related effects on sensitization in adulthood.

These data show that during adolescence, Poly I:C MIA increases reactivity over 30 trials in *Cntnap2*^–/–^ males, but this increase was not reflected in the sensitization score, suggesting that differences in baseline reactivity may have skewed the results. Moreover, *Cntnap2*^–/–^ offspring regardless of MIA, as well as MIA female offspring regardless of genotype, showed increased sensitization scores in adolescence. In adulthood, startle reactivity was increased due to complete *Cntnap2* knockout, similar to what was seen in the previous experiments, and *Cntnap2^+/–^* males showed an intermediate reactivity phenotype. *Cntnap2* knockout rats did not show increased sensitization, whereas Poly I:C MIA offspring, regardless of genotype, showed increased sensitization.

#### Pre-pulse inhibition

**FIGURE 5:**
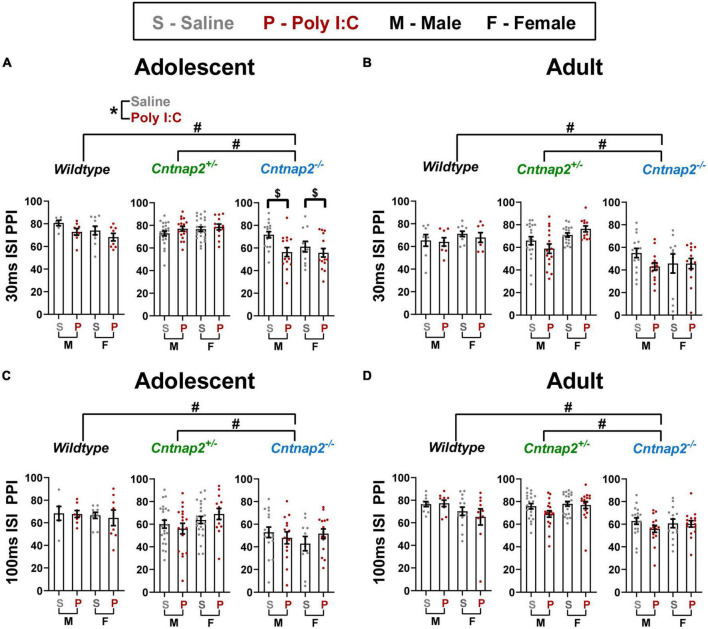
*Cntnap2* deficiency impairs PPI at all stimulus parameters, but Poly I:C MIA leads to further reduction in PPI in *Cntnap2*^–/–^ adolescent offspring with 30 ms ISIs. **(A)** Adolescent *Cntnap2*^–/–^ offspring show significantly reduced PPI at 30 ms ISI compared to both WT and *Cntnap2^+/–^* animals (adjusted *p* < 0.001; indicated by the ^#^ symbol), and Poly I:C MIA leads to further reduction of PPI in an independent manner across all genotypes (*p* = 0.013; indicated by the * symbol) and in a synergistic manner in *Cntnap2*^–/–^ males (*p* = 0.003; indicated by the ^$^ symbol). **(B)** In adulthood, Poly I:C MIA has no influence on PPI, but *Cntnap2*^–/–^ offspring still show greatly reduced PPI at 30 ms ISI (adjusted *p* < 0.001; indicated by the ^#^ symbol). **(C,D)** At 100 ms ISI, *Cntnap2*^–/–^ offspring show reduced PPI in both adolescence and adulthood (*p* < 0.001; indicated by the ^#^ symbol) and Poly I:C MIA does not influence PPI in any of the genotype groups. Data are shown as mean ± standard error. For information on sample sizes for each group, please see Table 1.

A repeated measures ANOVA was conducted at each age with pre-pulse intensity and ISI as within subject factors, and genotype, MIA, and sex as between subject factors. In adolescence, there was a significant ISI*genotype*MIA interaction [*F*_(2,155)_ = 4.447, *p* = 0.013]. To further investigate this interaction, 2 follow-up ANOVAs were conducted for each ISI. For 30 ms ISI, there were significant main effects of genotype [*F*_(2,155)_ = 30.001 *p* < 0.001; adjusted *p* < 0.001 for both *Cntnap2*^–/–^ vs. *Cntnap2^+/–^* and *Cntnap2*^–/–^ vs. WT; indicated by the ^#^ symbol in Figure 5A] and MIA [*F*_(1,155)_ = 6.247, *p* = 0.013; Poly I:C < saline; indicated by the * symbol in Figure 5A], as well as a significant genotype*MIA interaction [*F*_(2,155)_ = 6.054, *p* = 0.003]. *Post-hoc* testing revealed that Poly I:C *Cntnap2*^–/–^ offspring have decreased PPI compared to saline *Cntnap2*^–/–^ offspring (adjusted *p* = 0.001; indicated by the ^$^ symbol in Figure 5A). For 100 ms ISI, there was a main effect of genotype [*F*_(2,155)_ = 13.873], where *Cntnap2*^–/–^ offspring showed decreased PPI regardless of MIA (adjusted *p* < 0.001 for both comparisons; indicated by the ^#^ symbol in Figure 5C).

In adulthood, there was a significant ISI*genotype interaction [*F*_(1,141)_ = 41.149, *p* < 0.001] where *Cntnap2*^–/–^ offspring showed decreased PPI compared to *Cntnap2^+/–^* (adjusted *p* < 0.001) and WT (adjusted *p* < 0.001) offspring at both ISIs, but the extent of the decrease was much higher at 30 ISI (approximately 28% less PPI; indicated by the ^#^ symbol in Figure 5B) than at 100 ISI (approximately 7% less PPI; indicated by the ^#^ symbol in Figure 5D). Similarly, there was a significant genotype*sex interaction [*F*_(2,141)_ = 4.637, *p* = 0.011], whereby both sexes showed decreased PPI in *Cntnap2*^–/–^ offspring compared to *Cntnap2^+/–^* (adjusted *p* < 0.001) and WT (adjusted *p* < 0.001) offspring, but the extent of decrease in females was higher than that in males (approximately 21% decrease in females compared to 13.5% in males; data not shown).

Overall, PPI data show that Poly I:C MIA and *Cntnap2*^–/–^ genotype both independently and synergistically decrease PPI in adolescent male offspring. The *Cntnap2*^–/–^ genotype effect persists into adulthood, whereas the independent and synergistic Poly I:C MIA effects do not.

### Open field exploration

#### Total distance traveled

**FIGURE 6:**
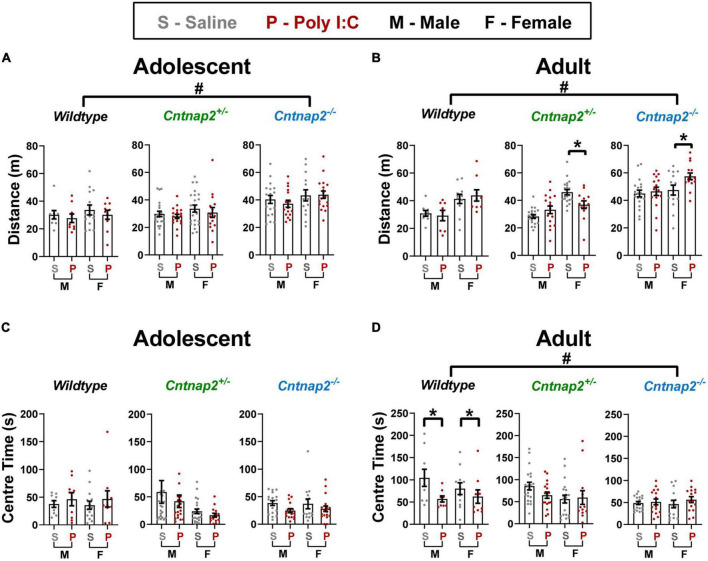
Poly I:C MIA influences locomotor activity and anxiety in the open field in a sex and genotype-dependent manner, whereas full *Cntnap2* deficiency induces changes in open field exploration in both adolescence and adulthood. **(A)** Adolescent *Cntnap2*^–/–^ offspring show hyperactivity measured by total distance traveled in the open field compared to WT and *Cntnap2^+/–^* offspring at 6 weeks of age (*p* < 0.001 for both comparisons; indicated by the ^#^ symbol). **(B)** In adulthood, *Cntnap2*^–/–^ hyperactivity persists (*p* < 0.001 for both comparisons; indicated by the ^#^ symbol), but Poly I:C MIA leads to reduced activity in *Cntnap2^+/–^* females (*p* = 0.019) and increased activity in *Cntnap2*^–/–^ females (0.013; both Poly I:C effects indicated by the * symbol). **(C)** Anxiety as measured by time spent in the center is not altered by either *Cntnap2* deficiency or Poly I:C MIA in adolescence. **(D)** In adulthood, Poly I:C WT offspring show reduced center time compared to WT saline offspring (*p* = 0.008; indicated by the * symbol), but Poly I:C MIA has no effects on *Cntnap2^+/–^* or *Cntnap2*^–/–^ offspring. Additionally, *Cntnap2*^–/–^ offspring show significantly decreased center time compared to WT offspring (*p* = 0.049 and *p* = 0.004, respectively; indicated by the ^#^ symbol). Data are shown as mean ± standard error. For information on sample sizes for each group, please see Table 1.

Here, we used the open field test to assess locomotor hyperactivity in saline and Poly I:C MIA offspring across age, sex, and *Cntnap2* genotypes by measuring the total distance traveled throughout the 30-min test session. A separate univariate ANOVA was conducted for each age with genotype, MIA, and sex as between-subject factors. In adolescence, there was a main effect of genotype [*F*_(2,175)_ = 18.363, *p* < 0.001], and *post-hoc* testing revealed a significant increase in total distance traveled in *Cntnap2*^–/–^ offspring compared to both *Cntnap2^+/–^* offspring (*p* < 0.001 for both comparisons, indicated by the ^#^ symbol in Figure 6A).

In adulthood, the ANOVA revealed a significant main effect of genotype [*F*_(2,159)_ = 31.767, *p* < 0.001; Figure 6B] and a significant genotype*MIA*sex interaction [*F*_(2,159)_ = 5.346, *p* = 0.006]. To further investigate this interaction, two follow-up ANOVAs for each sex revealed a significant genotype*MIA interaction in females only [*F*_(2,82)_ = 6.218, *p* = 0.003], where *Cntnap2^+/–^* Poly I:C females traveled less than *Cntnap2^+/–^* saline females (*p* = 0.019) and *Cntnap2*^–/–^ Poly I:C females traveled more than *Cntnap2*^–/–^ saline females (*p* = 0.013; both Poly I:C effects indicated by the * symbol in Figure 6B). In contrast, there was only a main effect of genotype in males [*F*_(2,83)_ = 25.346, *p* < 0.001] where *Cntnap2*^–/–^ males showed higher distance traveled compared to both *Cntnap2^+/–^* and WT males (*p* < 0.001 for both comparisons, indicated by the ^#^ symbol in Figure 6B). In summary, we confirmed that *Cntnap2*^–/–^ rats are hyperlocomotive, with mild and opposing effects of MIA in female adult *Cntnap2^+/–^* and *Cntnap2*^–/–^ rats.

#### Center time

Next, we tested the separate and combined effects of *Cntnap2* genotype, Poly I:C MIA, sex age on the time spent in the center of the open field, a rodent measure of anxiety behavior. A separate univariate ANOVA was conducted for each age with genotype, MIA, and sex as between-subject factors. In adolescence, there were no significant main effects or interactions associated with genotype or MIA (Figure 6C). In adulthood, there was a main effect of genotype [*F*_(2,159)_ = 6.040, *p* = 0.003], where *Cntnap2*^–/–^ animals spent significantly less time in the center of the open field compared to *Cntnap2^+/–^* and WT offspring (*p* = 0.049 and *p* = 0.004, respectively; indicated by the ^#^ symbol in Figure 6D). Additionally, there was a significant genotype*MIA interaction [*F*_(2,159)_ = 3.302, *p* = 0.039] and *post-hoc* testing revealed that WT Poly I:C offspring spent less time in the center of the open field compared to WT saline offspring regardless of sex (*p* = 0.008; indicated by the * symbol in Figure 6D).

### Social behavior in the 3-chamber test

For the habituation stage of the test, a repeated measures ANOVA was conducted to test for animal side preference before introducing the social stimuli. This ANOVA had side as within subject factor and genotype, MIA, and sex as between subject factors. In adolescence, there was a significant main effect of side, where animals generally preferred spending more time in the left chamber compared to the right chamber [*F*_(1,182)_ = 3.96, *p* = 0.048; average of 257 s in the left chamber compared to 241 s in the right chamber]. However, there were no significant interactions between side preference and any of the between subject factors. In adulthood, a similar ANOVA also showed a main effect of side, where animals generally preferred spending more time in the left chamber compared to the right chamber [*F*_(1,172)_ = 71.4, *p* < 0.001; 267 s in the left chamber compared to 218 s in the right chamber]. In addition, the ANOVA also showed an interaction between MIA and side [*F*_(1,172)_ = 4.719, *p* = 0.031], where Poly I:C animals preferred the left chamber slightly more than saline animals by spending approximately 11 s more in that chamber compared to saline animals, regardless of genotype or sex. Together, these data indicate that our experimental setup induced a left side preference in all animals. However, it is unlikely that this side preference influenced our sociability and social novelty results presented below given (1) the lack of interaction with genotype, MIA, and sex in adolescence, (2) the relatively small magnitude of the interaction with MIA in adulthood, and (3) the fact that we randomized side selection such that the placement of the social stimuli occurred with roughly the same probability in the left and right chambers.

For each of the sociability and social novelty tests, a repeated measures ANOVA was conducted at each age with side as a repeated factor and genotype, MIA, and sex as between subject factors. The social side is considered the one which control animals typically prefer, that is, the side with the conspecific in the sociability test and the side with the novel conspecific in the social novelty test.

**FIGURE 7:**
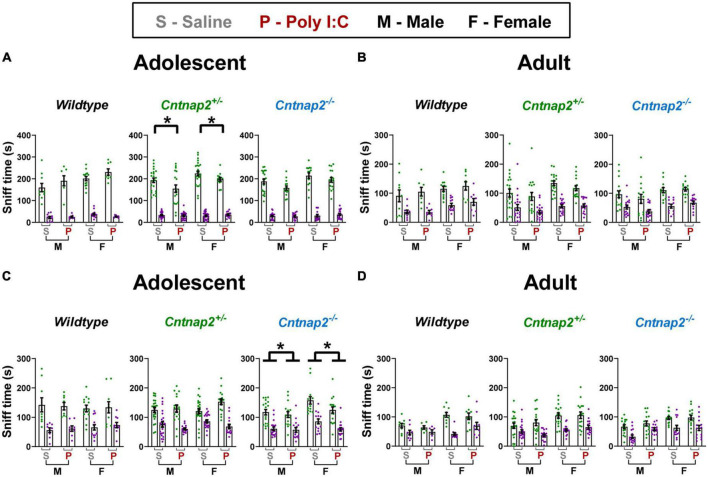
Poly I:C reduces social exploration but not social preference in adolescent *Cntnap2^+/–^ and Cntnap2^–/–^* offspring. In all groups at both ages, time spent in the social chamber/exploring the social animal holder was significantly more than the time spent in the non-social chamber/exploring the non-social animal holder, indicating that all groups showed strong preference for the social side/stimulus. **(A)** Poly I:C MIA reduced time spent sniffing the social stimulus in adolescent *Cntnap2^+/–^* but not WT or *Cntnap2*^–/–^ offspring (*p* = 0.013; indicated by the * symbol). **(B)** There were no significant effects associated with Poly I:C MIA or *Cntnap2* genotype in adult sociability exploration. **(C)** *Cntnap2*^–/–^ Poly I:C offspring spent significantly less time sniffing animal holders on both the social and non-social sides compared to their *Cntnap2*^–/–^ saline offspring. **(D)** There were no significant effects associated with Poly I:C MIA or *Cntnap2* genotype in adult social novelty exploration. Data are shown as mean ± standard error. For information on sample sizes for each group, please see Table 1.

For the sociability test, adolescent statistical testing showed a significant side*genotype*MIA interaction [*F*_(2,173)_ = 4.443, *p* = 0.003]. To follow up on this interaction, a separate analysis was conducted for each genotype which showed a significant side*MIA interaction for *Cntnap2^+/–^* offspring [*F*_(1,76)_ = 5.305, *p* = 0.024]. Together, these data show that Poly I:C *Cntnap2^+/–^* offspring spent less time sniffing the social animal holder compared to saline *Cntnap2^+/–^* offspring (*p* = 0.013; indicated by the * symbol in Figure 7A). In adulthood, there were no significant main effects or interactions associated with either genotype or MIA for time spent sniffing the social vs. non-social animal holders (Figure 7B). Overall, these data show that Poly I:C MIA reduces but does not abolish sociability preference in *Cntnap2^+/–^* offspring, which can be considered a relatively mild social deficit in this experimental protocol.

For the social novelty test, adolescent statistical testing showed a significant genotype*MIA effect [*F*_(2,170)_ = 3.445 *p* = 0.034] in the total time spent sniffing both social and non-social animal holders. *Post-hoc* tests revealed that Poly I:C *Cntnap2*^–/–^ offspring spent less time sniffing both the novel stranger animal holder and the familiar animal holder compared to saline *Cntnap2*^–/–^ offspring (*p* = 0.003; indicated by the * symbol in Figure 7C). In adulthood, there were no significant main effects or interactions associated with either Genotype or MIA for the time spent sniffing the social vs. non-social animal holders (Figure 7D). Overall, these data show that Poly I:C MIA reduces social novelty exploration time but not preference in adolescent *Cntnap2*^–/–^ offspring, which can be considered a mild social deficit in this experimental protocol.

## Discussion

The findings of this study demonstrate how genetic and environmental risk factors can act both independently and synergistically to alter ASD-related behavioral phenotypes in a sex and age-specific manner. The synergistic effects induced by Poly I:C MIA in *Cntnap2*^+/–^ and *Cntnap2*^–/–^ animals provide support for the gene-environment interaction hypothesis by showing that the genetic deficiency made animals more susceptible to the effects of the environmental insult. One of the primary goals of our study was to characterize the sensory processing phenotype of our double-hit model through startle phenotypes, which are known for their translatability across species (Koch, 1999; Swerdlow et al., 1999; Braff et al., 2001).

### Effects on startle reactivity and sensitization

Several studies have shown that autistic individuals exhibit increased reactivity to acoustic stimuli, even those as quiet as 65 dB (Kohl et al., 2014; Takahashi et al., 2014, 2018; Ebishima et al., 2019). Similarly, our group has shown that both the *Cntnap2*^–/–^ animals and Poly I:C MIA offspring show increased startle reactivity (Scott et al., 2018, 2020; Haddad et al., 2020a). We sought to extend these findings by attempting to decipher whether the increased reactivity is due to increased baseline startle or increased sensitization. Firstly, our results replicated the increased startle reactivity in *Cntnap2*^–/–^ offspring that has been associated with altered brainstem auditory processing and was ameliorated by systemic injection of the Gamma-Aminobutyric Acid (GABA)_*B*_ receptor agonist R-baclofen (Scott et al., 2018, 2020; Möhrle et al., 2021). In contrast, Poly I:C MIA did not alter startle reactivity, which conflicts with one of our previous findings using the same MIA protocol (Haddad et al., 2020a). This difference in results could be attributed to various factors that have been shown to alter susceptibility to MIA, including Poly I:C batch, rat strain, and housing conditions, all of which were different compared to our previous study (Schwartzer et al., 2013; Careaga et al., 2018; Morais et al., 2018; Mueller et al., 2018).

The startle reactivity findings strongly suggested that the increased startle response amplitude may be attributed at least partly to sensitization. Interestingly, our follow-up experiment that was designed to optimally detect sensitization showed that both *Cntnap2* knockout and Poly I:C MIA independently led to increased sensitization in an age and sex-specific manner. However, combined with the previous experiment, we concluded that only the increased reactivity in *Cntnap2* adolescent males was due to increased sensitization.

An important implication of these findings is that the same sensory processing phenotype—increased sensitization—can be caused by different pathophysiological mechanisms—Poly I:C MIA or *Cntnap2* deficiency—that manifest differently across age and sex. This could partly explain why it has been challenging to develop treatments for ASD, as therapeutics may not be able to simultaneously target multiple pathophysiological mechanisms (McCracken et al., 2021). Another implication of these findings is the importance of determining why an autistic individual develops sensory hyperreactivity. In a clinical setting, understanding whether hyperreactivity is due to an increase in baseline reactivity or an increase in sensitization can be used to more precisely inform the management of the disorder.

### Effects on pre-pulse inhibition

Decreased PPI has been reported in autistic individuals and many ASD animal models, including Poly I:C MIA offspring and *Cntnap2* knockout animals (Vuillermot et al., 2011; Meehan et al., 2017; Sinclair et al., 2017; Scott et al., 2018, 2020; Haddad et al., 2020b; Möhrle et al., 2021; Zheng et al., 2023). In line with these previous studies, both *Cntnap2* knockout and Poly I:C MIA led to decreased PPI. In addition, the two factors also interacted in adolescent offspring to further decrease 30 ms ISI PPI in *Cntnap2*^–/–^ offspring, indicating that a deficiency in *Cntnap2* leaves animals susceptible to Poly I:C MIA’s detrimental effects on sensorimotor gating.

The ISI-specific changes in PPI point toward specific brain structures and molecular mechanisms involved in the MIA*-Cntnap2*^–/–^ interaction that can be investigated in future studies. For instance, short ISI PPI (30 ms) is presumably mediated by projections from the ventral nucleus of the trapezoid body or the locus coeruleus to the cochlear root neurons, whereas longer ISI PPI (100 ms) is mediated by a more comprehensive circuit involving multiple brainstem nuclei that integrate multimodal pre-pulses (Gómez-Nieto et al., 2020). From a molecular perspective, it is hypothesized that PPI with short ISI is more dependent on the activation of ionotropic receptors, whereas PPI with longer ISIs depends on metabotropic neurotransmitter receptors (Jones and Shannon, 2000; Yeomans et al., 2010).

### Effects on open field exploration and social behavior

Hyperactivity is linked to both core symptoms and comorbidities in ASD. The DSM V diagnostic criteria for ASD include observations of repetitive patterns of behavior, which may manifest as hyperactivity (American Psychiatric Association, 2013). Furthermore, attention deficit hyperactivity disorder is a common comorbidity in individuals with ASD (Lai et al., 2019; Mutluer et al., 2022). Interestingly, locomotor hyperactivity measured in the open field test has been reported in both *Cntnap2*^–/–^ animals and Poly I:C MIA offspring (Peñagarikano et al., 2011; Tang et al., 2013; Zhu et al., 2014; da Silveira et al., 2017; Scott et al., 2020). On the other hand, anxiety is one of the comorbidities commonly associated with ASD (van Steensel et al., 2011; Hollocks et al., 2019; Lai et al., 2019; Mutluer et al., 2022) and was assessed in our study by measuring time spent in the center of the open field. Although *Cntnap2*^–/–^ animals have previously shown normal open field center time compared to controls, Poly I:C MIA is known to decrease center time in this paradigm, which is linked to increased anxiety (Meyer et al., 2005; Meyer, 2006; Brunner et al., 2015; Schaafsma et al., 2017; Kim et al., 2018).

Our purpose for including the social behavior tests follows from human studies associating atypical sensory processing across various modalities with social impairments (Thye et al., 2018). Similarly, past research has shown an association between sensory over-responsivity and anxiety, the latter being a common comorbidity in autistic individuals (van Steensel et al., 2011; Green et al., 2012; Hollocks et al., 2019). Lastly, hyperactivity could be linked to increased responsivity or impaired habituation to the sensory environment.

Contrary to our prediction, the results of the open field and social behavior tests showed a different pattern of effects compared to the startle experiments. For instance, unlike the genotype-independent increase in sensitization in adult Poly I:C MIA offspring, anxiety-like behavior was only increased in Wildtype but not *Cntnap2*-deficient MIA offspring. Upon further inspection, this discrepancy in the center time phenotype could be explained by a floor effect in *Cntnap2*^–/–^ animals. After all, complete *Cntnap2* knockout substantially reduced center time, possibly to a point that masks the effects of Poly I:C MIA.

In terms of social behavior phenotypes, our experiments showed that there were subtle gene-environment interaction effects that reduced sociability and social novelty exploration but did not abolish social preference. Combined with our other findings, these subtle differences indicate that sensory phenotypes measured through the acoustic startle response may not play a role in social exploration as measured by the 3-chamber test. For instance, direct social interaction where conspecific animals are free to move and physically interact provides a richer set of sensory stimulation than in our testing paradigm. In such a scenario, sensitization or impaired PPI could stand in the way of typical social interaction, which would not necessarily be captured in the restricted 3-chamber social apparatus. Indeed, our regular interactions with *Cntnap2*^–/–^ animals have shown that these animals are exceptionally alert and react excessively to handling by experimenters, even at the end of a 10-day experimental protocol during which they are handled daily.

Another key takeaway from both the locomotor hyperactivity and social behavior measures is the potential for a gene-environment interaction with *Cntnap2^+/–^* animals. This heterozygous genotype can be likened to a common gene variant that, on its own, offers a slight increase in the chance of developing ASD compared to rare variants that cause syndromic forms of ASD (Choi and An, 2021). Indeed, behavioral phenotyping has shown that *Cntnap2^+/–^* animals are comparable to Wildtype animals in many regards (Scott et al., 2020). An interaction effect with Poly I:C MIA suggests that this partial genetic deficiency could increase susceptibility to environmental insults such as maternal infection during pregnancy (Clarke et al., 2009; Meyer, 2019). Interestingly, Poly I:C MIA interactions with the *Cntnap2^+/–^* genotype present differently compared to those with *Cntnap-/-* genotype, as seen by our locomotor hyperactivity results.

### Experimental considerations

#### Immune response variability

An important consideration in Poly I:C experiments is the extent of the maternal immune response that is elicited by the mother. In this experiment, the maternal immune response was confirmed through the detection of increased IL-6 and TNF-α 3 h after Poly I:C MIA. However, the cytokine response was highly variable between dams. Given that the same batch and dose of Poly I:C was used throughout our experiment, this variability could be attributed to our use of dams from four different unrelated genetic backgrounds, each with a unique immune profile that could have altered the course of the immune response mounted against Poly I:C.

#### Breeding scheme

Our experiment utilized a rather unique breeding scheme by generating all the litters using one male breeder and female breeders from four different families unrelated to each other and the male breeder. ASD has a strong hereditary component and paternal characteristics like age and genetic/epigenetic profiles are known to influence the risk for various neurodevelopmental disorders including ASD (Vervoort et al., 2022). More specific to this field of study, the effects of MIA are likely dependent not just on the maternal immune signals, but also on the fetal response to those signals, which could be influenced by immune-related genes passed on from the father. Therefore, to reduce the impact of paternal confounding variables and more precisely study the effects of MIA on offspring behavioral phenotypes, we decided to use the same breeding male. To our knowledge, this type of breeding scheme has not been previously performed in a Poly I:C MIA study, although it is difficult to evaluate such a claim given the paucity of breeding information provided in the majority of Poly I:C MIA studies.

## Conclusion

Altogether, our study’s findings support the double-hit gene-environment interaction hypothesis of ASD by showing that Poly I:C MIA interacts with both partial and complete *Cntnap2* deficiency to alter offspring behavior. By using the acoustic startle response and its modulations to measure sensory processing phenotypes, our findings can be directly translated into other preclinical ASD models and human studies. Future studies can explore the structural and functional brain correlates of these behavioral findings to locate vulnerable brain networks or molecular pathways impacted simultaneously by multiple ASD risk factors. Despite showing several double-hit interactions, it is important to consider that each risk factor also had independent effects on offspring phenotypes, which were at times additive in nature. This serves as a reminder that even if overlapping mechanisms between different risk factors are discovered, the effects of each genetic alteration or environmental exposure must still be considered in the context of ASD diagnosis, treatment, and management.

## Data availability statement

The original contributions presented in this study are included in the article/Supplementary material, further inquiries can be directed to the corresponding author.

## Ethics statement

This animal study was reviewed and approved by the Animal Care Committee, University of Western Ontario.

## Author contributions

FH conducted experiments, analyzed data, made figures, and wrote the first draft of the manuscript. CD has helped with data acquisition and edited the manuscript. SS, FH, and CD conceptualized the study. All authors contributed to the article and approved the submitted version.

## Footnotes

1
BioRender.com
