# Supplementary material for: Investigating behavioral phenotypes related to autism spectrum disorder in a gene-environment interaction model of Cntnap2 deficiency and Poly I:C maternal immune activation
Source: Front Neurosci. 2023 Mar 14;17:1160243. doi: 10.3389/fnins.2023.1160243 (PMC10043204; doi:10.3389/fnins.2023.1160243)
Supplement: Supplementary file 1 [file Image_1.pdf]

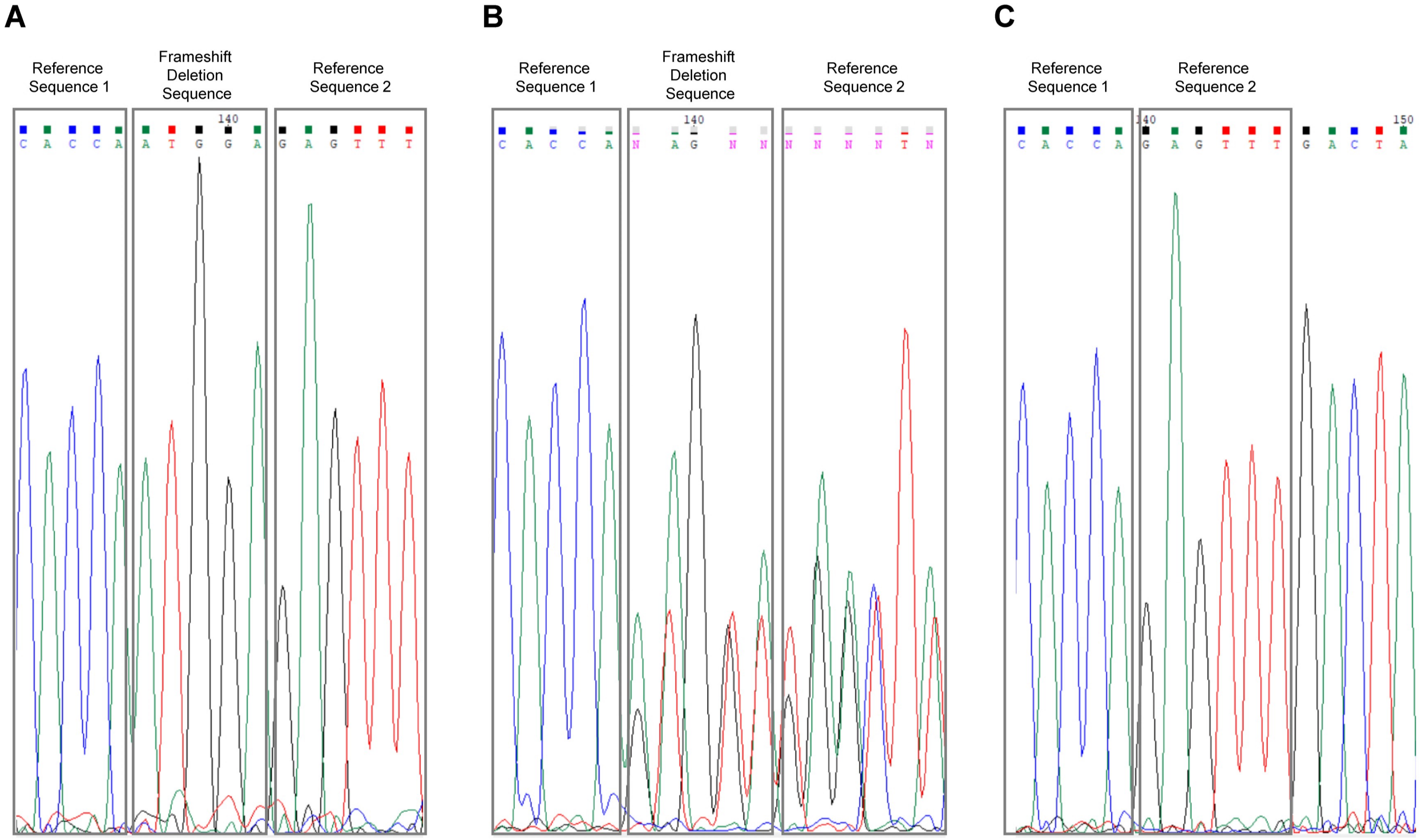

SUPPLEMENTARY FIGURE 1
Representative sequencing data showing the Cntnap2 gene sequence in Wildtype (A), Cntnap2+/- (B), and Cntnap2-/- (C) animals. The 5-base-pair frameshift deletion is highlighted, along with the 5-base-pair sequence preceding the deletion (Reference Sequence 1) and the 6-base-pair sequence following the deletion (Reference Sequence 2).
